# Abscess of the Iliac Fossa

**Published:** 1842-07-02

**Authors:** 


*Abscess of the Iliac Fossa.*—M. Barthélemy recommends in cases of extra-peritoneal abscess of the iliac fossa, the careful and repeated examination of the lumbar region, in order that, if positive indications of the collection of pus can be discovered there, an opening may be made, rather than allow the matter to make its way into the pelvis, where its presence may cause death. He adds, that the opening of abscesses there is neither difficult nor dangerous, inasmuch as the abscess itself interposes between the abdominal parietes and the peritoneum. After the operation the patient should be kept lying on his back, and a tent be passed into the opening, to be replaced next day by emollient injections.—*Annales de Chirurgie.*

